# Structural, morphological, dielectric, electric and magnetic properties of ${\text{Ba}}_{1-x}{\text{Dy}}_{2x/3}{\text{Ti}}_{0.98}{\text{Mn}}_{0.02}O_{3}$ ceramics for multifunctional applications

**DOI:** 10.1016/j.heliyon.2024.e32505

**Published:** 2024-06-08

**Authors:** S.A. Mamun, Mithun Kumar Das, K.S. Uddin, T. Ahamed, Mohammad J. Miah

**Affiliations:** a Department of Physics, Comilla University, Cumilla, 3506, Bangladesh; b Uttara University, Dhaka, Bangladesh

**Keywords:** Perovskite, Dielectric properties, Impedance, Magnetic properties

## Abstract

Herein, the standard solid-state reaction process was employed to synthesize the polycrystalline Ba_1-x_Dy_2x/3_Ti_0.98_Mn_0.02_O_3_ (*x* = 0.0000–0.0085) ceramics and each composition was sintered at 1200 °C for 3 h. The structural, morphological, electrical, and magnetic properties were carried out by the X-ray diffraction (XRD), field emission scanning electron microscopy (FESEM), impedance analyzer, and vibrating sample magnetometer (VSM) to investigate the influence of doping of *Dy*^*3+*^ (low concentration) and Mn^4+^ in BaTiO_3_ simultaneously. The XRD study confirmed the formation of perovskite structure with tetragonal symmetry of the prepared solid solution. The magnitude of the porosity (P%) decreased from 13.22 to 9.49 with increasing content of *Dy* and *x* = 0.0080 sample showed the lowest value. The mean grain size was estimated in the micrometer range, with values ranging from 0.5713 to 0.1457 μm. The highest grain size determined for the *x* = 0.0070 sample was 0.5713 μm. The Brunauer-Emmett-Teller (BET) adsorption isotherm measurements were used to estimate the specific surface area; the result was 24.181 m^2^/g for x = 0.007 composition. For the compound with x = 0.0070 the maximum recorded dielectric constant was found to be 6 × 10^3^ at 10^3^ Hz. A relatively lower dielectric loss (<5 %) was observed. The Nyquist plot illustrated that only the grain boundary effect is significant for the conduction process in the studied compositions. The present solid solution revealed better magnetic results compared to other reported ceramics similar to the prepared constituents. The optimum value of saturation magnetization (0.371 emu/g) was obtained for *x* = 0.0080 composition. Among the synthesized *Dy* doped samples *x* = 0.0075 composition displayed a significant complex initial permeability ($\mu_{i}^{/}$). An enhanced relative quality factor (RQF) was seen with increasing frequency and the highest relative quality factor was noticed (>100) for the *x* = 0.0075 sample at 10^8^ Hz. The studied materials could be employed as an environmentally acceptable alternative to the hazardous lead (Pb)-based multiferroic substance.

## 1 Introduction

Ferroelectric crystals offer stable and switchable electric polarization in the form of accommodating atomic displacements. They have strong dielectric properties and exhibit a polarization versus electric field hysteresis loop. Titanate series ceramics are a novel class of ceramics with ferroelectric properties, developed in recent years. The most promising of them is BaTiO_3_ (BTO), which demonstrates exceptional ferroelectric, piezoelectric, and dielectric properties [1]. For technological systems based on electro-ceramics and microelectronics, such as capacitors, thermistors, high-storage memory devices, semiconductors, piezoelectric devices, and ultrasonic transducers, this is a popular choice [2]. It is utilized in ferroelectric direct access memory (FRAM), multilayer ceramic capacitors, positive temperature coefficient thermistor rings, ultrasonic detectors, dielectric waveguides, multilayer substrates, microwave integrated circuit substrates, piezoelectric sensors, optoelectronic devices, actuators, and more [3,4]. A large number of research groups in the globe are also working with another ceramic-like material, such as ferrite. Ferrites have numerous desirable properties, including strong electrical resistivity, low losses over a wide range of frequencies, high mechanical hardness, chemical and environmental stability, low production costs, and so on, which make them useful in a variety of electronic devices [[5], [6], [7], [8]]. In recent past, most of the research work was done on the mixed ferrites, which are synthesized by the mixture of divalent metal ions both in tetrahedral and octahedral sites. The cation distribution of the mixed ferrite has a significant impact on the ferrites' surface properties, making them catalytically active. However, a number of scientists have attempted to improve the material's various properties by swapping the host cations within the perovskite structure of BaTiO_3_ (BTO). By changing the corresponding cation for A (Ba-site) or B (Ti-site), BTO's electric and dielectric characteristics can be tailored [9]. Various kinds of impurity atoms can be hosted within the perovskite structure of BTO, however, the appropriate doping at the A- or B-site mostly depends on the dopants' atomic radius, valence electron, and the nature in order to offset the defect mechanism [10]. A relatively little quantity of dopant inclusion can cause a phase transition, which can change the dielectric and electrical properties by modifying the crystallite structure, increasing the grain size, and altering the microstructure's homogeneity [11]. The rare earth element *Dy*^*3+*^(Dysprosium) or *Gd*^*3+*^(Gadolinium*)* can be a good choice as dopant impurities, since many scientists have reported the effect of rare earth dopants on the dielectric, as well as magnetic properties in BTO [[12], [13], [14]]. The doped BTO ceramics exhibit better results but still it has some drawbacks, for example, leakage current, impurities, and low resistance characteristics. The leakage current can limit the creation of dielectric and ferroelectric properties and also its widespread applications. Moreover, when a significant quantity of dopant is introduced into BTO, the oxygen vacancies are originated and, thus, it loses its insulating property and works as a semiconductor [15].

Despite being a great insulator, BTO also shows semi-conductivity with the doping of trivalent or pentavalent rare earth elements. It was reported in numerous investigations that the trivalent elements, like La^3+^ with higher atomic radii occupy the Ba^2+^ site, while the pentavalent elements Nb^5+^ occupy at the Ti^4+^ site, resulting in access positive charge, which is a common behavior of the donor atom. Therefore, the electronic compensation is needed to operate it due to the donor-doping mechanism, which results in the reduction of Ti^4+^ and switching to Ti^3+^. The higher donor dopant contents lead to more compensation through the electronic mechanism with the creation of greater cation vacancies. Later research, based on both defect energy calculations and experimental data, demonstrated that titanium tetra-ionized vacancies are the most likely compensatory defects in the heavily donor-doped BaTiO_3_. A. Ianculescu et al. [15] reported the other findings that the switch of ionic towards electronic charge compensation induced by decreasing La content is unlikely to occur. Still, reduced La-doped BaTiO_3_ ceramics could have semiconducting behavior associated with small amounts of oxygen loss during their sintering at higher temperatures ≥1350 °C. Lowering donor content and reducing the sintering temperature for the prepared ceramic samples could be typically used as insulators and semiconductors. The ions from the middle of the rare-earth series, such as Yb^3+^, Er^3+^, Dy^3+^, and Sm^3+^ show amphoteric behavior and can occupy both cationic lattice sites in the BaTiO_3_ structure [[16], [17], [18]].

Although certain theoretical calculations suggest that, over a wide range of intermediate ionic radii, the trivalent impurities will split equally into the two sites to generate donor-acceptor compensation. Still, there is a question if the concentration is lowered and the temperature remains below 1350 °C what effects will happen for these amphoteric ions doping, like Yb^3+^, Er^3+^, Dy^3+,^ Gd^3+^, and Sm^3+^? But the literature results on the simultaneous substitution of both A-site and B-site of the BTO ceramics and a thorough study on the structural, electrical, and magnetic properties are very few. An effort has been made to monitor the influence of *Dy*^*3+*^ and the transition metal *Mn*^*4+*^ on the mentioned properties of BTO. In this research article the behavior of lower amounts of *Dy*^*3+*^ (*x* = 0.0000, 0.0070, 0.0075, 0.0080, and 0.0085) doping and sintered at 1200 °C on microstructure, dielectric, electric, and magnetic nature are illustrated in great detail.

## 2 Experimental details

### 2.1 Sample preparation

The solid solution of Ba_1-x_Dy_2x/3_Ti_0.98_Mn_0.02_O_3_ (*x* = 0.0000, 0.0070, 0.0075, 0.0080, and 0.0085) ceramics were manufactured utilizing a typical solid-state reaction process using the raw materials BaCO_3_ (99.99 %), Dy_2_O_3_ (99.99 %), TiO_2_ (99 %), and MnO_2_ (99 %). According.

To the stoichiometric formula the raw materials were taken in an agate mortar. Table 1 shows the amount of all reagents used to synthesis various composition of Ba_1-x_Dy_2x/3_Ti_0.98_Mn_0.02_O_3_ ceramics. Each composition was milled for 6 h to ensure a homogenous mixture. After that, the mixture of each composition was calcined at 900 °C for 3 h in the furnace. The mixed powder samples were grinded again for roughly 4 h after calcining. By combining polyvinyl alcohol (PVA) with the weighted powder, disc- and toroid-shaped materials were formed, which were obtained using the hydraulic press at 5 tons of uniaxial pressure. Finally, the prepared samples have been sintered for 3 h at 1200 °C.

**Table 1:** Calculation of raw materials used to synthesis Ba_1-x_Dy_2x/3_Ti_0.98_Mn_0.02_O_3_ (*x* = 0.0000, 0.0070, 0.0075, 0.0080, and 0.0085) ceramics.

Composition	Amount of BaCO_3_ (g)	Amount of Dy_2_O_3_ (g)	Amount of TiO_2_ (g)	Amount of MnO_2_ (g)
BaTiO_3_	3.38	–	1.36	–
Ba_0.9930_Dy_0.0047_Ti_0.98_Mn_0.02_O_3_	3.363	0.0146	1.3457	0.0268
Ba_0.9925_Dy_0.00507_Ti_0.98_Mn_0.02_O_3_	3.36	0.0160	1.3458	0.0268
Ba_0.9920_Dy_0.0053_Ti_0.98_Mn_0.02_O_3_	3.35	0.0169	1.3459	0.0268
Ba_0.9915_Dy_0.0056_Ti_0.98_Mn_0.02_O_3_	3.34	0.0179	1.3460	0.0268

### 2.2 Experimental technique

The crystal structure of the prepared compound was studied using the X-ray diffractometer (Rigaku, Model: Smart Lab) with CuK_α_ radiation (λ = 1.5406 Å) at room temperature and 2° per min scanning speed in a range of 2θ from 20 to 80°. A voltage of 40 kV and a current of 40 mA were used in the experiment. The data obtained from the XRD was utilized to calculate the lattice parameters. To identify the structural stability, the tolerance factor (*t*) of the sintered material was determined by the Gold-Schmidt formula [19] in equation (1):

(1) $$t=\frac{R_{A}+{\;R}_{O}}{R_{B}+R_{O}}$$

where, *R*_*A*_, *R*_*B*,_ and *R*_*O*_ are the ionic radii of A-site atom, B-site atom, and oxygen atom, respectively, in the ABO_3_ perovskite structure. For the synthesized Ba_1-x_Dy_2x/3_Ti_0.98_Mn_0.02_O_3_ ceramics the value of *t* was evaluated using equation (2):

(2) $$t=\frac{\left(1-x\right)R_{{\text{Ba}}^{2+}}+\frac{2x}{3}R_{{\text{Dy}}^{3+}}+{\;R}_{O^{2-}}}{\sqrt{2}\left[0.98R_{{\text{Ti}}^{4+}}+0.02R_{{\text{Mn}}^{2+}}+R_{O^{2-}}\right]}$$

where *R*_*Ba*_^*2+*^, *R*_*Dy*_^*3+*^, *R*_*Ti*_^*4+*^, *R*_*Mn*_^*2+*^, and *R*_*O*_^*2−*^ are the ionic radii of the elements *Ba*, *Dy*, *Ti*, *Mn,* and *O*, respectively. The samples' bulk density (ρ_*B*_*)* was measured by the Archimedes method using (3), (4):

(3) $$\text{Volume}\;\text{of}\;\text{the}\;\text{sample}=\frac{\;\text{mass}\;\text{of}\;\text{the}\;\text{sample}\;\text{in}\;\text{air}\;‐\;\text{mass}\;\text{of}\;\text{the}\;\text{sample}\;\text{in}\;\text{water}}{\text{density}\;\text{of}\;\text{water}}$$

(4) $$\text{Samples}’\;\text{bulk}\;\text{density}\;\left(\rho_{B}\right)=\frac{\text{mass}\;\text{of}\;\text{the}\;\text{sample}\;\text{in}\;\text{air}}{\text{volume}\;\text{of}\;\text{the}\;\text{sample}}$$

The theoretical density (ρ_x_) of the samples was determined by equation (5),

(5) $$\rho_{x}=\frac{n\times M_{A}}{N_{A}V}\text{,}$$

where, *n* is the number of atoms in a unit cell, *M*_*A*_ is the molar mass of the sample, *N*_*A*_ is the Avogadro's number, and *V* is the volume of the unit cell. The following equation (Eq^n^. 6) was used to calculate the porosity (P) of the synthesized compound:

(6) $$P=\left(1-\frac{\rho_{B}}{\rho_{th}}\right)\times 100\%$$

The microstructure of the sintered samples was examined by the Field Emission Scanning Electron Microscopy (FESEM, model no. JEOL JSM 7600F). Talos f200 × was used for Transmission Electron Micrographs (TEM). The Brunauer-Emmett-Teller (BET) adsorption isotherm measurement (Model: BET-201-A) was employed to obtain the specific surface area of the crystalline powders.

The dielectric measurements were carried out at room temperature within the frequency range of 1000Hz–10^8^ Hz by using an Impedance Analyzer (Wayne Kerr 6500B). To measure the dielectric properties the samples were painted on both sides by conducting silver paste to ensure good electrical contacts. The dielectric constant ($\varepsilon^{/}$) was calculated from the capacitance using equation (7):

(7) $$\varepsilon^{/}=Ct/\varepsilon_{0}A$$

where, C is the capacitance of the pellet, A is the cross-sectional area of the electrode, and ε_0_ (=8.85 × 10^−12^ F/m) is the permittivity in free space. The complex electric modulus (M*) was evaluated by relating the real part of dielectric constant ($\varepsilon^{/}$) to the imaginary part ($\varepsilon^{//}$) by using the following equations (8), (9):

(8) $$M^{/}=\frac{\varepsilon^{/}}{\varepsilon^{/2}+{\;\varepsilon }^{//2}}$$

(9) $$M^{//}=\frac{\varepsilon^{//}}{\varepsilon^{/2}+\varepsilon^{//2}}$$

where, M^/^ and M^//^ are the samples' real and imaginary part of electric modulus (M*). The dielectric modulus was obtained from the complex impedance analysis spectroscopy, and the relationship is,

M^/^ = 2*πfε*_o_*Z*^//^ and M^//^ = 2*πfε*_o_*Z*^/^

Where, *Z*^/^ is the real part and *Z*^//^ is the imaginary part of the complex impedance. The AC conductivity (*σ*_*ac*_) of the samples was calculated using equation (10):

(10) σ_ac_ = ωε_o_ε^/^tanδ_E_

Where, ω is the angular frequency and tan*δ*_*E*_ is the dielectric loss. The M − H hysteresis loops were collected at room temperature by the Physical Properties Measurement System (PPMS) and the Quantum Design Dyna Cool to reveal the magnetic properties of the resulting materials. The magnetic properties of the compound were also discussed through the complex initial permeability. The real part ($\mu_{i}^{/}$) and imaginary part ($\mu_{i}^{∕∕}$) of the complex initial permeability (μ_i_*) were measured as a function of frequency within the range of 1 kHz to 10^8^ Hz using the Wayne Kerr 6500B Impedance Analyzer. The values of $\mu_{i}^{/}$ and $\mu_{i}^{∕∕}$ were perceived using the following equations (Eq^n^. 11 and 12):

(11) $$\mu_{i}^{/}=\frac{L_{s}}{L_{o}}$$

(12) $${\text{and}\;\mu }_{i}^{∕∕}=\mu_{i}^{/}\;\tan\;\delta_{M}$$

where, L_S_ is the self-inductance of the sample core and L_0_ is the inductance of the winding of the coil without the sample and tanδ_M_ is the magnetic loss. L_0_ was derived from the geometrical relations, L_0_ = μ_0_N^2^S/π$\bar{d}$, where, μ_0_ is the permeability in vacuum, N is the number of turns of the coil (N = 4), S is the cross-sectional area, and $\bar{d}$ =(d_1_+d_2_)/2 is the mean diameter of the toroid-shaped sample, where, d_1_ and d_2_ are the inner and outer diameter of the toroid-shaped sample, respectively.

## 3 Results and discussion

### 3.1 XRD analysis

**Fig. 1:**
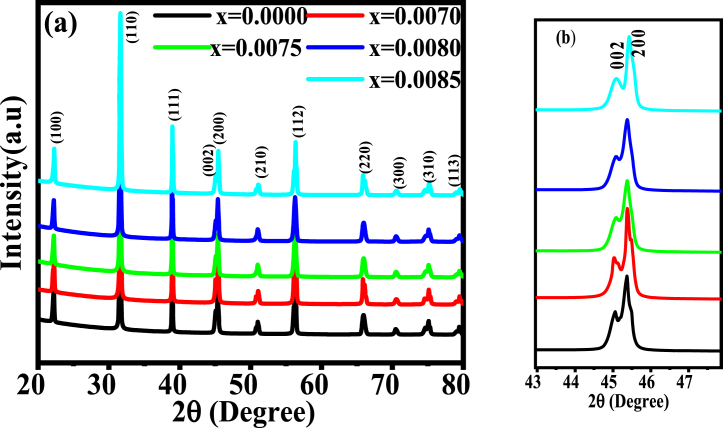
(a) X-Ray Diffraction pattern for various ${\text{Ba}}_{1-x}{\text{Dy}}_{2x/3}{\text{Ti}}_{0.98}{\text{Mn}}_{0.02}O_{3}$ (b) Enlarged view of peak position between 43 and 47°.

One of the most popular techniques for examining the structural characteristics of solids is the X-ray diffraction (XRD) method. This method is widely used to identify different phases of generated perovskite, as well as unit cell parameters to define the basic crystal structure and other parameters, like density, porosity, and tolerance factor. Fig. 1(a) and (b) display the XRD patterns for the prepared Ba_1-x_Dy_2x/3_Ti_0.98_Mn_0.02_O_3_ (BDTMO) compositions and enlarged view of peak position between 43 and 47°, respectively. Observing more conscientiously it is clear that the profound peaks in the XRD of the specimens seem to suit augmentations of inter planer distance, changes of peak intensity, and the peak shift towards the lower or higher reflection angle [20]. However, the lattice parameter was determined by the atomic size, specifically the atomic radii of the dopant and substituted atoms [21]. The magnified scale of the diffraction pattern for every composition within the 2θ range of 44 to 46° clearly shows there are two peaks (002) and (200). The presence of double peaks, instead of single peak one, suggests the tetragonal symmetry for the prepared compounds from *x* = 0.0000 to *x* = 0.0085. The single reflection peak corresponds to cubic perovskite structure.

The XRD pattern obtained for the prepared compositions are in good agreement with the standard XRD pattern of BaTiO_3_ particle (JCPDS card no. 79–2264) [22] and this type of structural phase formation has also been reported by others [23,24]. There is no significant impurity peak for the *Dy* doping other than the tetragonal structures, which represents the homogeneity of the studied samples. The peak positions of the *x* = 0.0085 sample were observed to migrate towards greater 2-theta, indicating a reduction in an inter planner spacing, which, in turn, results in the increase in lattice strain and shrinking the crystal structure. For *x* = 0.0070 the prominent peak has been split into two peaks (101) and (110). The rest of the peaks does not shift much towards lower or higher 2θ angle. The lattice parameter (*a* and *c*) has been calculated corresponds to tetragonal structure. Aliovalent ion substitution causes the crystal shift resulting in the increase of oxygen vacancy from cubic to tetragonal or tetragonal to hexagonal for further more oxygen vacancy [25].When more *Dy* is added, the lattice parameter *a* is found to decrease and the decreasing character of *a* with increasing *Dy* concentration might have been caused due to the larger ionic radius of Dy^3+^ than that of Ba^2+^ [26].

### 3.2 Lattice parameter and crystallite size measurement

**Fig. 2:**
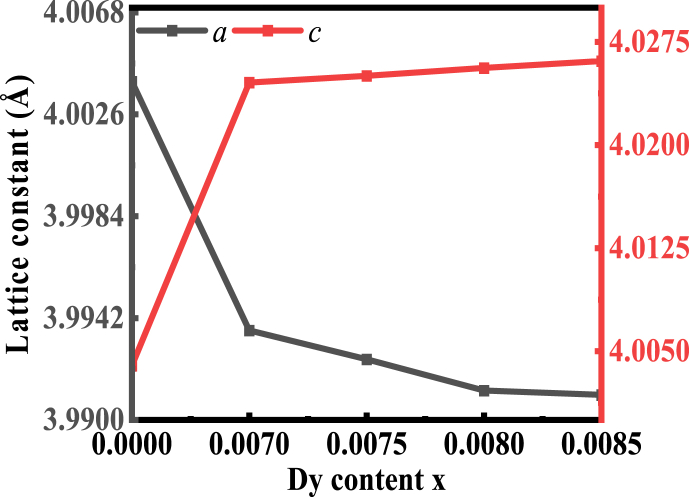
The variation of *a* and *c* with Dy doping.

Fig. 2 shows the changing variation of lattice parameter *a* and *c* with *Dy* content while the values of *a* and *c*. are listed in Table 2. With increased *Dy* at A-site with a fixed amount of *Mn* at the B-site there is a slight contraction of lattice parameter *a*, while there is a slight elongation of *c*. The lattice constant and the ionic radius of the cations at the tetrahedral sites are related. As previously stated, if the radius of the substituted cations is greater in size the unit cell expands, and the lattice constant increases [27].

**Table 2:** The values of *a*, *c,* cell *v*olume, *σ*, *t*, *ρ*_*B*_, *ρ*_*th*_, and *P%* of BDTMO ceramics.

*Dy* content (x)	a (Å)	c (Å)	a^2^ × c (Å)^3^	σ	t	***ρ***_***B***_***g/cm***^***3***^	***ρ***_***th***_***g/cm***^***3***^	P (%)
0.0000	4.00395	4.00395	64.1898	3.9271	0.9336	5.7100	6.0316	5.33
0.0070	3.99369	4.02455	64.1898	4.5498	0.9319	5.2324	6.0294	13.22
0.0075	3.99250	4.02503	64.1592	3.9298	0.9317	5.4263	6.0322	10.04
0.0080	3.99121	4.02561	64.1269	3.3234	0.9316	5.4618	6.0347	9.49
0.0085	3.99103	4.02611	64.1291	3.1458	0.9314	5.4563	6.0339	9.57

So, due to the ionic size difference between *Ba*^*2+*^ and *Dy*^*3+*^ with higher contents of *Dy* a slight decrease in *a* and an increase in *c* was obtained. When *Dy* is doped into the crystal structure of barium titanate, it introduces an ionic size mismatch. *Dy* ions have a larger ionic radius compared to both barium and titanium ions. The larger *Dy* ions tend to occupy the interstitial sites within the lattice. As a result of this ionic size mismatch and the occupation of interstitial sites, two main effects are observed: one is a decrease in *a.* The reason of this is the larger *Dy* ions exert compressive strain on the lattice, causing a reduction in the distance between the adjacent Ti atoms (reduction in *a*). Another one is an increase in *c*. Introduction of *Dy* ions can lead to an increase in the *c* lattice parameter. This is because the larger *Dy* ions occupying the interstitial sites cause an expansion in the vertical axis, resulting in an increased distance between the adjacent *Ba* atoms (increase in *c*). The lattice parameters (*a* and *c*) acquired for the current study has been compared with other available literature values related to our synthesized composition. The *a* and *c* values of the reported compositions have been presented in Table 3. The obtained result is found to be identical to the published values.

**Table 3:** The comparison of the published lattice parameter with the present investigation.

Ceramics	Lattice Parameter		Refs.
	*a* (Å)	*c* (Å)	
BaTiO_3_	3.9947	4.0336	[35]
Ba_1-x_Dy_2x/3_TiO_3_ (x = 0.0–0.1)	3.996–3.999	4.030–4.010	[36]
BaTi_1-x_Mn_x_O_3_ (x = 0.0–0.1)	3.994–3.998	4.022–4.029	[37]
Ba_1-x_Sm_x_Ti_0.99_Mn_0.01_O_3_ (x = 0.02–0.07)	3.988–4.005	4.005–4.033	[38]
Ba_1-x_Dy_2x/3_Ti_0.98_Mn_0.02_O_3_ (x = 0–0.0085)	3.9910–4.003	4.0039–4.0245	Present work

**Fig. 3:**
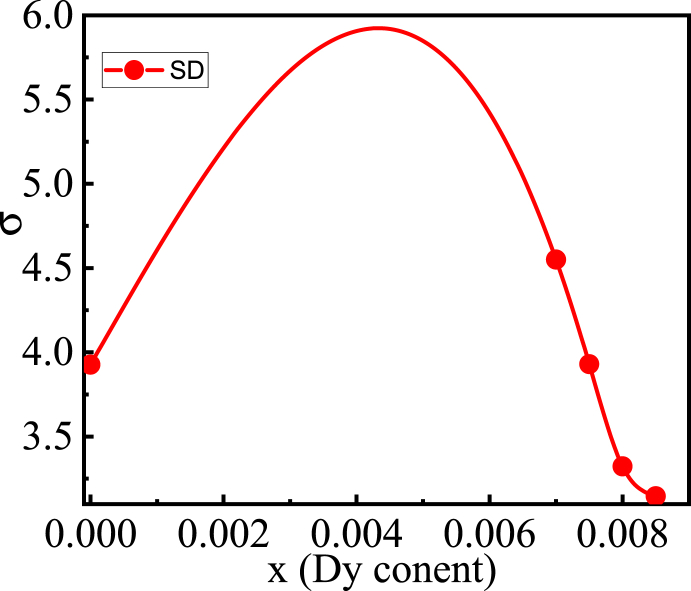
The variation of standard deviation with different *Dy* contents.

The crystallite sizes of the Dy doped barium titanate nano powder, calcined at 900 °C for 3 h, were estimated following the Debey-Scherrer's equation [28]: $D=\frac{0.94\lambda }{\beta \;\cos\;\theta }$, where, D is the crystallite size, λ is the wavelength of CuK_α_ (=1.5406 Å), β is the FWHM in radian, and θ is the Bragg angle. The standard deviation of the attained crystallite size has been determined using the relationship: $\sigma =\sqrt{\sum \frac{{\left(x_{i}-\bar{x}\right)}^{2}}{n-1}}$, where, $x_{i}$ is the value in the data distribution, $\bar{x}$ is the mean of crystallites, and n is the total number of observations. The values of standard deviation (σ) of the crystallite size is listed in Table 2 and the variation of σ, with *Dy* content, has been noticed in Fig. 3. The estimated value of σ is found within the range from 3.1458 to 4.5498. The highest σ, determined for the sample *x* = 0.0070, is 4.5498 and the lowest one (3.1458) is obtained for *x* = 0.0085, which indicate more deviation among crystallites exist in *x* = 0.0070 sample, and after that the deviation is reducing with the addition of *Dy.* This signifies that the sorting of crystallites improves with the addition of *Dy* concentration, where the crystallites of *x* = 0.0085 composition are very well sorted and a poorly sorted crystallites is noticed in *x* = 0.0070 compound.

The stability and geometrical shape deviation of perovskite from the ideal cubic structure are measured by the tolerance factor (*t*). The standard value of *t* is considered 0.77 to 1.1. For every composition, the value of *t* is calculated using the Gold-Schmidt formula. It was noticed that *t* decreases with increasing *Dy* concentration, with values ranging from 0.9336 to 0.9314, as shown in Table 2. The reason for decreasing *t* can be attributed to smaller ionic size of *Dy*^*3+*^ than *Ba*^*2+*^ as mentioned earlier [29], i.e., the size difference between the substituent and substituted ions can cause the variation of *t*.

### 3.3 Density and porosity study

**Fig. 4:**
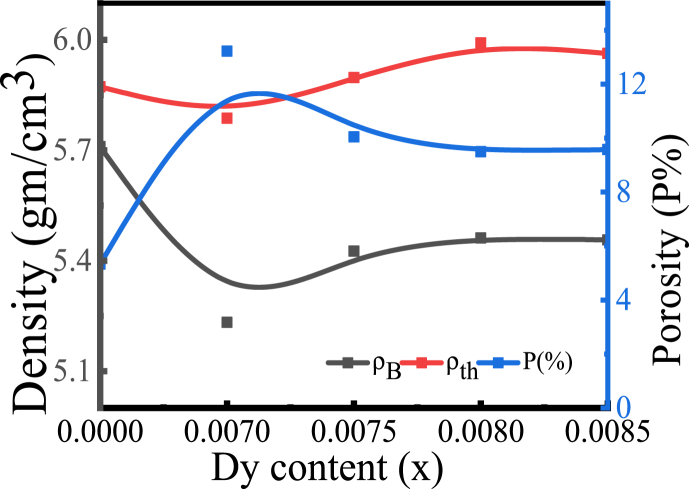
The variation of *ρ*_*B*_, *ρ*_th_, and *P* for the concentration of *Dy* in BDTMO.

The generation of vacancies for heat evaporation, the creation of pores in the materials, and several other parameters, including crystallite size, and cell volume, all affect the density. The change in bulk density (*ρ*_*B*_), theoretical X-ray density (*ρ*_*th*_*),* and porosity (*P%),* as a function of *Dy* content, is shown in Fig. 4, and the magnitudes for *ρ*_*B*_, *ρ*_*th*_, and P (%) are listed in Table 2. The value of *ρ*_*B*_ is calculated according to the Archimedes method and the values are obtained ranging from 5.4618 to 5.2324 g/cm^3^ with *Dy* content. The highest *ρ*_*B*_, determined for the sample *x* = 0.0080, is 5.4618 g/cm^3^.The variation of *ρ*_*B*_ is mostly due to the variation of dopant density. The larger the dopant molecular density the higher is the *ρ*_*B*_ [30]. Despite of higher molecular density of substituent *Dy* (162.998 g/mol) than the substituted Ba (137.3268 g/mol) the value of *ρ*_*B*_ experienced a decrease and this is because of the formation of some oxygen deficiencies in the samples [31]. Furthermore, another cause of the decrease of *ρ*_*B*_ in each sample after doping, is that the microstructure contains intergranular porosity resulting from the higher rate of grain growth [32,33]. For *x* = 0.0070, the value of *ρ*_*B*_ is the lowest (5.23 g/cm^3^) containing the maximum pores, as shown in the SEM image.

Fig. 4 demonstrates, *ρ*_*th*_ is higher than *ρ*_*B*_, and the reason behind this is that the samples may have pores that were originated during the sintering process, as well as the modifications to the element, which alter the interatomic distance, leading to create pores [34]. The magnitude of the porosity (P%) has reduced from 13.22 to 9.49 with increasing *Dy* dopant, and *x* = 0.0080 sample displayed the lowest value, as shown in Fig. 4. The porosity can result from two sources, including intragranular and intergranular porosity that is P = P_intra_ + P_inter_ and the intergranular porosity may be responsible for the prepared samples.

### 3.4 Surface morphology and EDX study

The microstructure of a material has a significant impact on its bulk characteristics and performance. A comprehensive analysis of microstructures includes characterizing the size, shape, distribution, and composition of grains and second-phase particles, as well as the defect structures, which are sometimes overlooked. Unquestionably, the most important microstructural factor controlling the thermal, mechanical, electrical, magnetic, and other properties of polycrystalline materials is the grain size. Therefore, the material grain size ought to be taken into account when choosing materials for engineering purposes.

The FESEM images at magnification of × 10^3^ and the excitation of 30 kV electron beam have been shown in Fig. 5(a). The grain development is clearly detected in the samples *x* = 0.0000 and *x* = 0.0070, despite the fact that the grains in the parent samples are abnormal. The average grain size from SEM images was calculated for each composition using the Image J software. Fig. 5(b) exhibits histogram for the grain distribution of each composition. The average grain size ($\bar{D}$) was obtained 0.4521, 0.5713, 0.1567, 0.1826, and 0.1457 μm for *x* = 0.0000, 0.0070, 0.0075, 0.0080, and 0.0085, respectively. The value of $\bar{D}$ was found to increase for 0.07 % *Dy*, with further doping of *Dy* $\bar{D}$ was found to decrease. The reason behind the increase in $\bar{D}$ is an increase of oxygen vacancy and the decrease in $\bar{D}$ is due to lowering of oxygen vacancy [29]. Due to the size discrepancy between the dopant and substituent atoms, the grain size is shown larger [39]. The grain size shows reducing trend during higher addition of *Dy*, which signifies that the rise in *Dy* ion concentration in BaTiO_3_ ceramics inhibits the grain growth. One probable explanation for this phenomenon is the charge compensation mechanism that results from the valence difference between *Dy*^*3*+^ and *Ba*^*2*+^, which causes cation vacancies to form. The increase in internal compressive stress could be another significant indicator. This stress suppressed the spontaneous deformation of the unit cell and, thus, forced the formation of cubic phase and decreased tetragonality.

**Fig. 5(a):**
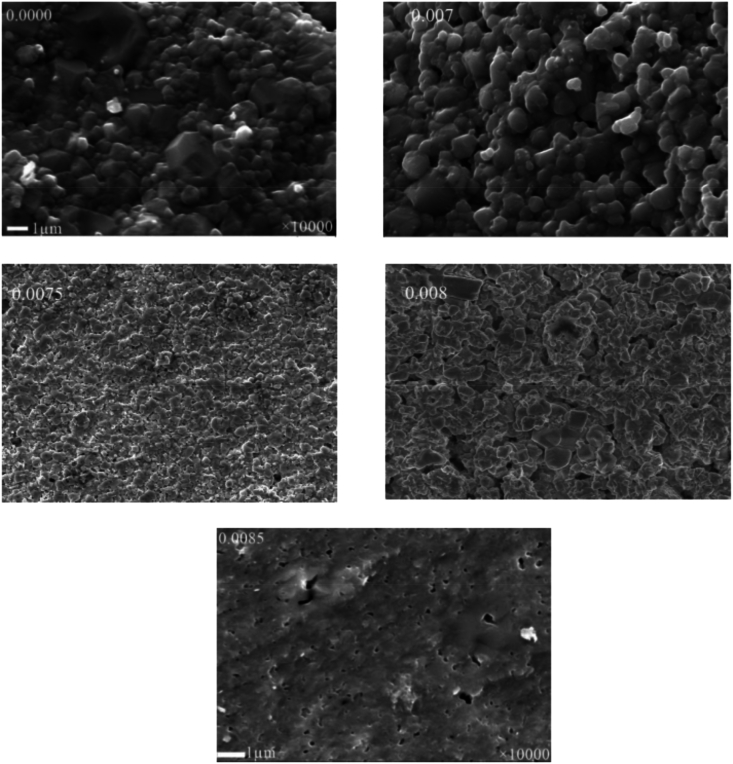
FESEM images for various fractured BDTMO polycrystalline ceramics.

**Fig. 5(b):**
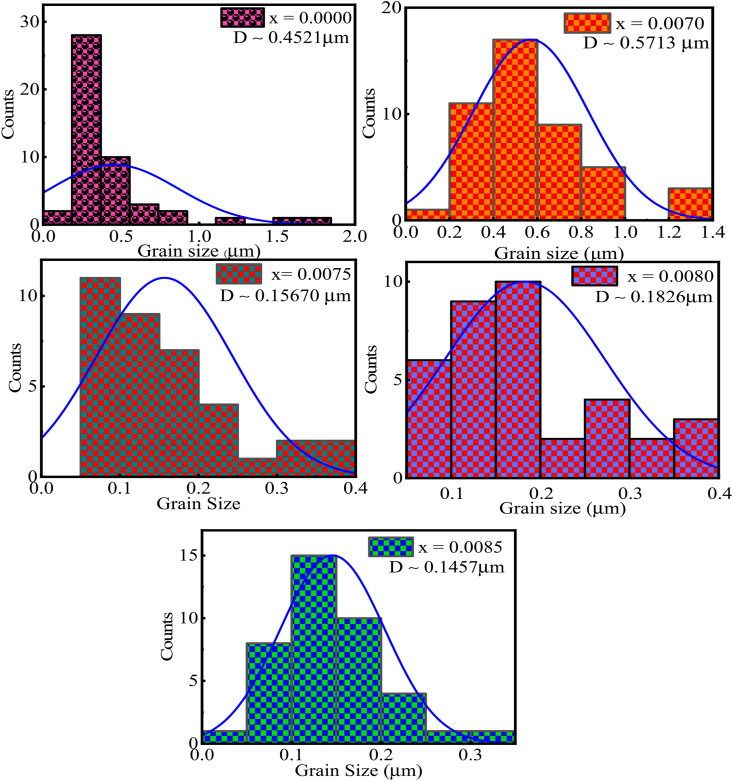
The histograms for the grain distribution of BDTMO compositions.

The Elemental Dispersive X-ray (EDX) spectra (Fig. 5(c)) of Ba_0.9930_Dy_0.0047_Ti_0.98_Mn_0.02_O_3_ (*x* = 0.0070) powder calcined at 900 °C for 3 h was carried out to identify the elemental composition of the produced compound. The presence of Ba, Dy, Ti, Mn, and O are noticed in the spectra. Some impurities of Cu are also presented in the spectra. The concentration (wt. %) of Ba, Dy, Ti, Mn, and O are 15.70, 0.24, 20.062, 0.49, and 27.062, respectively. This provides further evidence that the conditions of the preparation are totally satisfactory to the manufacturing of mixed oxides.

**Fig. 5(c):**
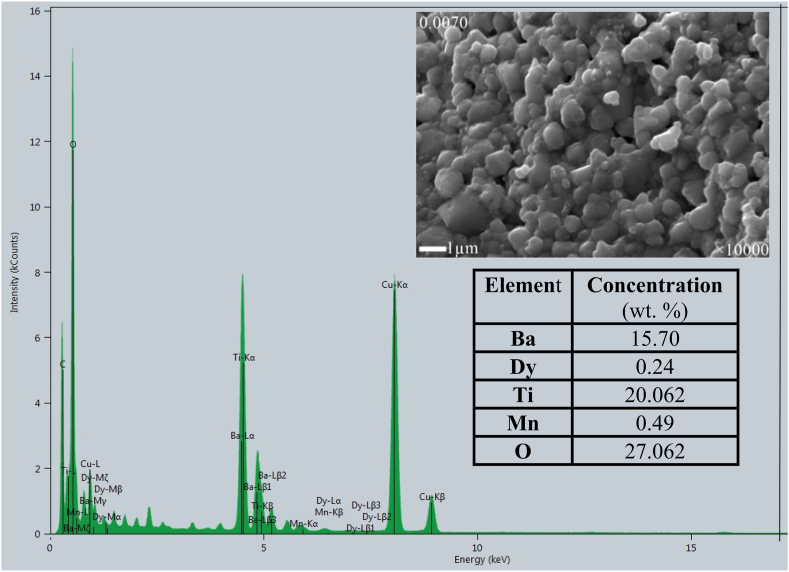
The EDX spectra of *x* = 0.0070 compositions with the elemental count (wt %).

### 3.5 Transmission Electron Microscope (TEM)

The most significant physical attribute of a particle is its size, which has a direct impact on materials several properties like dielectric characteristics, permeability, reactivity, features *etc*.

**Fig. 6:**
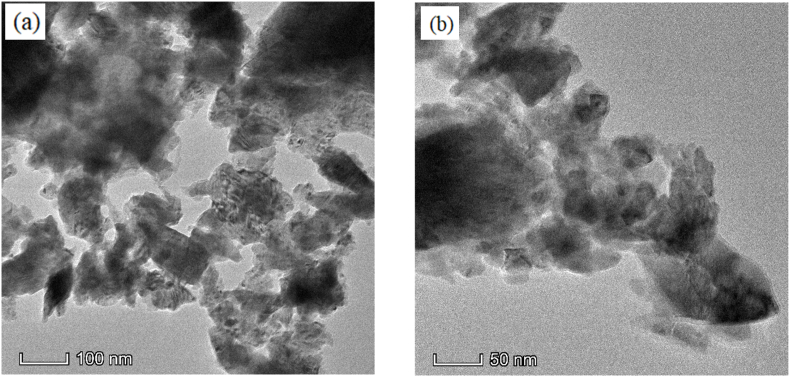
TEM images for (a) 100 nm magnification and (b) 50 nm magnification of Ba_0.9930_Dy_0.0047_Ti_0.98_Mn_0.02_O_3_ (*x* = 0.0070) ceramics.

Fig. 6 (a and b) illustrates the TEM of Ba_0.9930_Dy_0.0047_Ti_0.98_Mn_0.02_O_3_ (*x* = 0.0070) composition. The rate of reaction increases as the particle size decreases because the reactant's surface area increases. On the other hand enhance permeability is obtained for a bigger size particle. To calculate particle size of the manufactured solid solution Transmission Electron Micrographs (TEM) has been carried out. The TEM image shows that the nanoparticles are nonuniform and rod -shaped with curved ends projected. It is seen of Fig. 6 that many intermediate primary crystallites, they are joined together to form large crystals. Particle size of the doped sample is determined using the obtained TEM by ImageJ software. Average particle size is found to be 13.43 nm.

### 3.6 Brunauer-Emmett-Teller (BET) analysis

The surface area of solids is regarded as an important parameter that provides information on the voids present on the solid surface, which also influences the rate of chemical reactions. In general, the rate of chemical reaction within a substance is increased when the surface area of the substance is increased. The specific surface area of Ba_0.9930_Dy_0.0047_Ti_0.98_Mn_0.02_O_3_ (*x* = 0.0070) ceramics was determined by the Brunauer-Emmett-Teller (BET) adsorption isotherm measurements and the value was found 24.181 m^2^/g. The Brunauer-Emmett-Teller (BET)

**Fig. 7:**
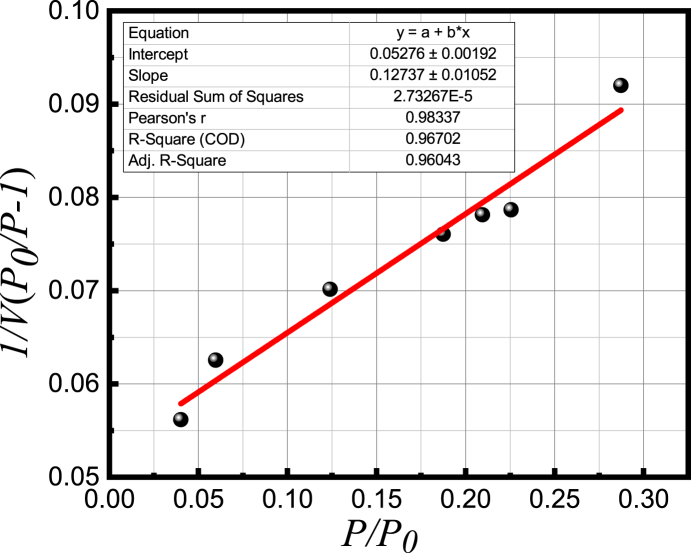
The $1/V\left(P_{0}/P-1\right)$*vs.*$P/P_{0}$ graph for *x* = 0.0070 composition.

Analysis is the most widely standard method for calculating the surface area of solids and powders. The following formula was applied to estimate the value of specific surface area: *SSA* = $\frac{v_{m}\;N\;s}{V}$, where, *N* is the Avogardo's number, *s* is the adsorption cross section of the adsorbate, *V* is the molar volume of the adsorbate gas, and $v_{m}=\frac{1}{\text{slope}+\text{intercept}}$ is the units of volume, which are also the units of the monolayer volume of the adsorbate gas. To determine the value of $v_{m}$ the magnitudes of slope and intercept were collected from the $1/V\left(P_{0}/P-1\right)$ *vs.* $P/P_{0}$ graph (Fig. 7). The estimated value of the specific surface area is consisted with the previous results of the research presented in Ref. [40]. The value that was obtained for BaTiO_3_ was 25.275 m^2^/g.

### 3.7 Dielectric constant measurements

**Fig. 8:**
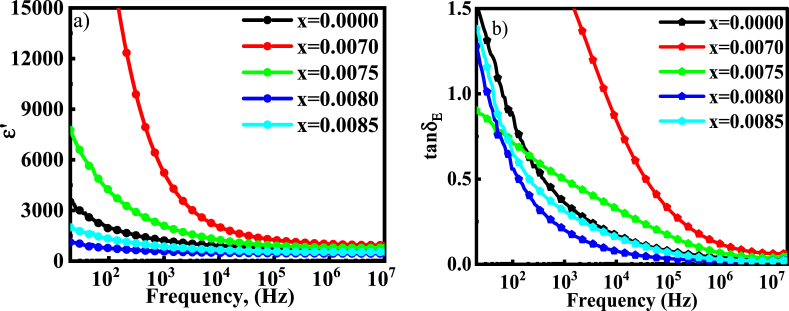
The variation of (a) dielectric constant and (b) dielectric loss for various BDTMO ceramics.

The dielectric constant is a qualitative and quantitative measurement of polarizing ability of a material with applied field and corresponds to the capability of storing electrical energy as potential energy and storing charge due to polarization. This is a complex quantity containing real (ε^/^) and imaginary (ε^//^) part and is expressed as [41], ${\;\varepsilon }^{*}=\varepsilon^{/}-j\varepsilon^{∕∕}$. The ε^/^ is represented as the effectiveness of the dynamical screening effect caused by the charge excitations at a particular frequency in a system. The frequency dependency of the dielectric constant for several BDTMO ceramics at 20Hz to 10^7^ Hz is shown in Fig. 8(a). The dielectric constant ($\varepsilon^{/}$) for all samples has followed a normal pattern al low frequencies, with a higher dispersion rate comparing to that of higher frequency range. At higher frequencies, it finally approaches a pattern that is almost constant, as was reported earlier [42]. This phenomenon is crucial and general for most ferroelectric materials, and the existence of all types of polarization, such as electronic, ionic, dipolar, and interface polarization, is the cause for a substantial dielectric constant at low frequency range [43]. Furthermore, the high values of ε^/^ at low frequencies are due to the aggregation of dislocation at the interface, oxygen vacancies, and defects at grain boundaries [44]. Another reason is that the space charge quickly obeys the fluctuation of the applied field at the low frequency range and becomes static at higher frequency, and the disappearance of all types of polarization, except the one observed in case of the electric polarization, happens due to the inability to respond quickly to the imposed electric field [45]. At low frequency ε/is different for each sample; this is because of the formation of heterogenous grain and grain boundary for different doping levels, besides higher ε/at lower frequency is because of an accumulation of electron at the grain boundary, resulting the polarization to happen [46].

The presence of an accessible charge carrier, which may not have had enough time to develop polarization at high frequency, causes the space charge to polarize dramatically. As a result, the available charge carrier goes through a relaxation mechanism and, eventually, *ε*^*/*^ approaches a constant value. With the increased amount of *Dy* concentration, the ε^/^ first increases, and, then, there is a state of decline. The maximum value of ε^/^ (≈ ${\;6\times 10}^{3}$) was obtained for *x* = 0.0070 and the minimum for *x* = 0.0085 (as shown in Fig. 8). This conclusion is attributed to the fact that substituting *Ba*^*2+*^ at the A-site with *Dy*^*3+*^ causes defects due to the generation of electron vacancies or barium vacancies in the crystal lattice, as both of which can produce a substantial lattice distortion in order to maintain an electric neutrality. When a tiny amount of *Dy* (low concentration) is supplied initially, it may operate as an effective dopant, enabling improved polarization behavior and, hence, a rise in the dielectric constant. The presence of *Dy* can introduce additional defect dipoles and increase the overall polarization response of the material. The studied compound is a BaTiO_3_-based ceramics. The properties of BaTiO_3_-based ceramics depend on many factors, such as composition, stoichiometry, impurity content, average crystallite size, grain size distribution, porosity, crystalline anisotropy, and sintering conditions. Undoped BaTiO_3_ exhibits insulating behavior and, traditionally, it is used as a ferroelectric material. When high amount of dopant is included in BaTiO_3_ it loses its insulating behavior and, then, it works as a semiconductor [15]. Moreover, oxygen vacancies are originated and, thus, the dielectric properties are deteriorated. When BaTiO_3_ is doped with low concentration, the vacancy of oxygen is reduced and its properties is found to improve, on the basis of dopant type. A large number of research group has long been worked doping with lower amounts of rare earth elements in BaTiO_3_ and noticed various changes in the properties [[16], [17], [18]]. The dielectric properties are found to be improved when Ba-site is doped by rare earth elements or alkaline metals and Ti-site is doped by the transition metals. In the current compositions, low amount (<0.1 at %) *Dy* is inserted in Ba-site, as well as Mn (fixed amount) is added in the Ti-site. Because of doping *Dy* and *Mn* ions, simultaneously, a modification in the cell volume, density, and microstructure is observed, which may be attributed to change in the ε^/^. However, as the concentration of *Dy*^*3+*^ increases beyond a certain critical point, it may cause undesirable interactions between the dopant ions. This can inhibit the turning ability of electric domains, which in turn reduces the turning direction polarization of a certain number of electrical domains. Additionally, this can lead to the formation of defects, impurities, or charge carriers, which can diminish the overall polarization response and cause the dielectric constant decrease [47].

Fig. 8(b) displays the change in *Dy* doping loss tangent (tan*δ*_*E*_) with different frequencies at room temperature. The dielectric loss tangent is seen to follow the same pattern as the dielectric constant. The absorption current can be produced at low frequencies by a variety of factors, including impurities, defects, and pores in the composite. The absorption current dropped as the frequency rose. The impact of material defects is minimal at high frequency. Thus, the dielectric losses are comparatively small [48]. All samples exhibit a dispersion in tan*δ*_*E*_ at lower frequencies and, especially, predominant at *x* = 0.0070, where the values vary from 4.8022 to 0.0801. The loss tangent of doped barium titanate initially increases due to the increase in defect generation and the charge carrier scattering was caused by the *Dy* impurity, i.e., the cation redistribution between the tetrahedral or octahedral site. However, higher *Dy* concentrations reduce the loss tangent due to compensating and saturation effects of charge distribution. Similar result was also reported earlier for *Zn* substituted nickel ferrite [49].

### 3.8 Electric modulus

**Fig. 9:**
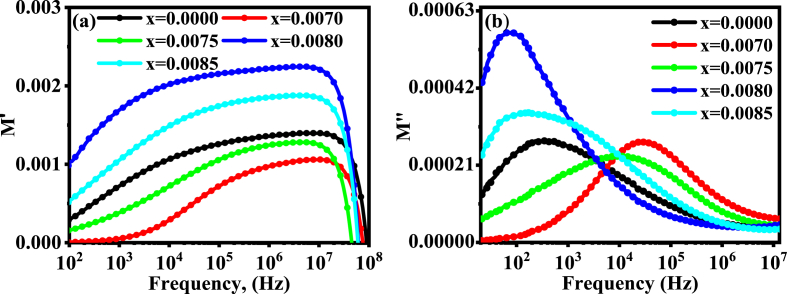
The variation of (a) real ($M^{/}$) and (b) imaginary ($M^{∕∕}$) parts of dielectric modulus for the BDTMO ceramics.

Fig. 9(a) and (b) show the modulus spectra for both the real ($M^{/}$) and imaginary ($M^{∕∕}$) parts. As can be seen from the figure, $M^{/}$ has a very low value in the lower frequency zone and rises with increasing frequency until a saturation value for BDTMO is reached. The short-range charge carrier mobility may contribute to a frequency range's propensity to attain saturation by promoting the conduction process at higher frequencies [50]. Another cause is the lack of restoring force at MHz frequency range [51]. The low value of $M^{/}$ at lower frequencies demonstrates the ease of polaron hopping and the tiny contribution of the electrode effect [52]. The value of $M^{/}$ increases with frequency and asymmetrically approaches the saturation with *Dy* content; this could be due to lower sensitivity and response of materials to the applied oscillating electric field at higher frequency. The smaller quantity of $M^{/}$ at lower frequency is attributed due to the mobility of the charge carrier over long distances.

The saturation state for all samples, however, has not orderly changed with *Dy* concentration. The maximum value of $M^{/}$ has been found for the sample with *x* = 0.0080 indicating the highest restoring force for this concentration. The value of $M^{∕∕}$ (imaginary part of modulus) shows different peak at different frequencies for various concentrations of *Dy,* the reason goes to different relaxation times at different frequencies. The presence of a lower frequency peak in the imaginary part of the dielectric modulus indicates that ions can go great distances, whereas the presence of a high frequency peak indicates that ions are constrained in their potential well [53]. The relaxation peak shifts towards the higher frequency for the lowest *Dy* doping concentration *x* = 0.0070 that is the relaxation rate is very low for *x* = 0.0070, and the relaxation rate is higher for *x* = 0.0080. However, the region below the peak frequency determines the extent to which charge carriers are long-range mobile, while above the peak frequency, carriers are trapped in a potential well and the short-range mobility is apparent. The region where the peaks occur indicates a transition from the long-range mobility to short-range mobility [54].

### 3.9 AC conductivity

**Fig. 10:**
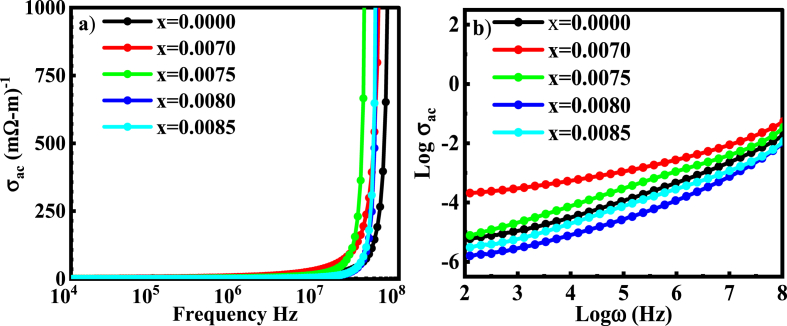
The variation of (a) *σ*_*ac*_ and (b) log*σ*_*ac*_ with frequency for various BDTMO ceramics.

The ac conductivity is an eminent measurement system to describe various conduction processes and the magnetic properties in any material, as electrical properties, are executed by the conductivity. The change in ac conductivity (*σ*_*ac*_), as a function of frequency, is seen in Fig. 10(a). The *σ*_*ac*_ executed by polaron capability greatly depends on the amount of independent charge carrier available in the composite. The ac conductivity is dominated by the polaron mechanism resulted from the polarizing effect or distortion effect in the neighboring lattice due to the mobility of free charge carrier as an instantaneous response to the applied electric field [55].

Larger polaron hoping leads to decrease the ac conductivity with frequency, but smaller polaron hoping σ_ac_ significantly rises according to frequency [56]. The frequency-dependent σ_ac_ spectra in the figure show two distinct sections. The grain boundary effect is heavily dominant in the low-frequency range, where the conduction is frequency-independent and is known as the *σ*_*dc*_ conductivity. At higher frequency region, typically known as the hopping region, *σ*_*ac*_ rises more rapidly than *σ*_*dc*_. This is due to the fact that when frequency increases, the grains in the hopping zone become more attentive, which causes the hopping charges to rise instantly in reaction to the increased frequency and, ultimately, causes an increase in *σ*_*ac*_ [57]. In the current study, it was revealed that *σ*_*ac*_ increases linearly for all samples of *Dy* doping with increasing frequency. This indicates that the coupling's conduction mechanism has small polaron hopping. The varying value of log*σ*_*ac*_ as a function of log*ω* is seen in Fig. 10(b). In all samples, the value of log*σ*_*ac*_ increases as the frequency increases, indicating that the material's conduction process is produced by small polaron hopping, which results in linearity in the log*σ*_*ac*_ variation and fits the Jonscher's power law.

### 3.10 AC resistivity

**Fig. 11:**
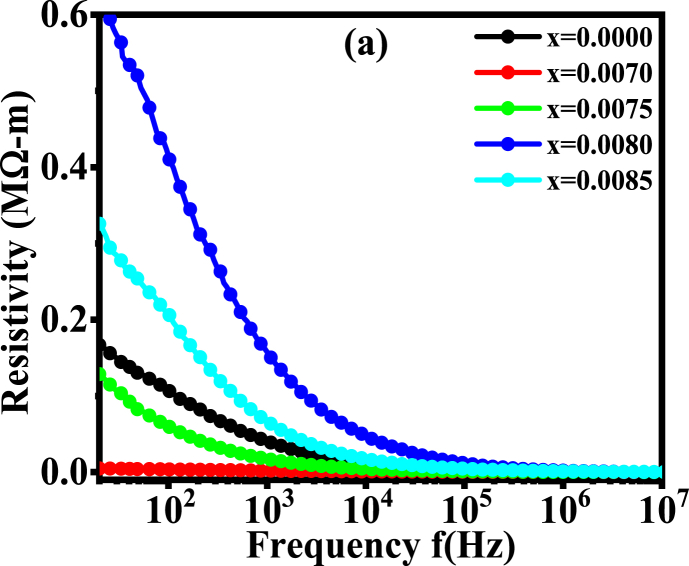
The variation of resistivity as a function of frequency for various BDTMO ceramics.

The resistance behavior of the studied materials is shown in Fig. 11. For all compositions, the resistivity (*ρ*_*ac*_*)* is found to be decreased with the increase in frequency.

The maximum resistivity is seen for *x* = 0.0080 *Dy* content, whereas the lowest resistivity is obtained for the composition *x* = 0. 0070. This implies that higher doping of *Dy* would result in higher resistivity, that is, the lowest conductivity would be found. However, it is noticeable that the dopant amount of *Dy* is significantly very low which are really different from thousands of conventional researches. Moreover, the variation of resistance and conductivity is not inconsiderable, which leads to a point that very small amount of doping can cause considerable change in the electric properties of barium titanate ceramics.

### 3.11 Complex impedance study

**Fig. 12:**
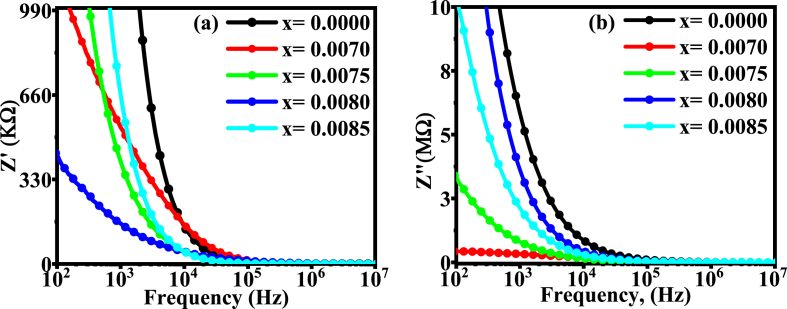
The variation of (a) $Z^{/}$, and (b) $Z^{∕∕}$ with respect to frequency for different BDTMO ceramics.

The variation of real part of impedance $\left(Z^{/}\right)$ with frequency is depicted in Fig. 12(a). The values of impedance $Z^{/}$ decreases dynamically at lower frequency, then, approaches to a certain static state over a wide range of frequency, indicating an increment of carrier conductivity up to a certain limiting frequency and, then, follow the frequency independency nature.

The fluctuation of $Z^{∕∕}$ with frequency follow the same trend as the variation of $Z^{/}$, as illustrated in Fig. 12(b). A greater value of impedance at a lower frequency indicates the presence of a higher polarization rate due to the presence of all types of polarization in the sample. However, the magnitudes of $Z^{/}$ and $Z^{∕∕}$ merge at very high frequency for all samples, and approaches a low value due to probable discharge or accumulation of space charge at homogeneous phase boundaries under the applied electric field [55]. At greater frequency levels, the lack of space charge is responsible for lowering the impedance and, hence, suppressing further polarization [58]. Moreover, the absence of any peak in the $Z^{/}$ vs. frequency and $Z^{∕∕}$ vs frequency graphs indicates a non-Debye type dielectric relaxation [59].

### 3.12 Nyquist plot

**Fig. 13:**
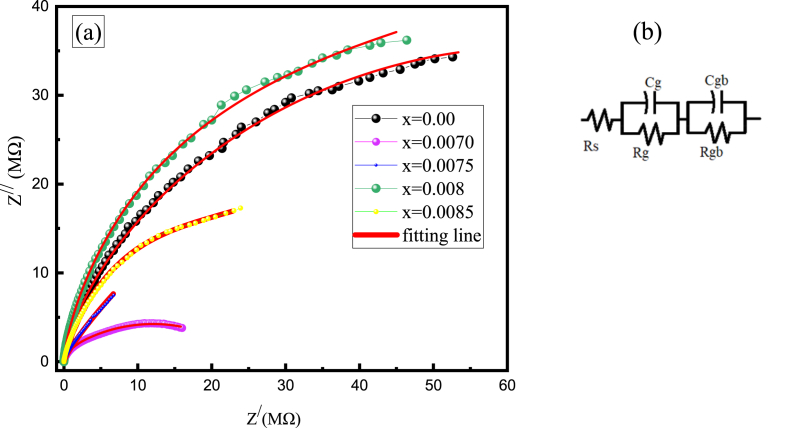
(a) The Nyquist plot for various BDTMO ceramics and (b) equivalent RC circuit.

The quantitative and qualitative dominance of grain and grain boundary effects on the polycrystalline materials is commonly assessed using the impedance spectroscopy. Based on the electrical properties, there could be two semicircular arcs in the $Z^{∕∕}$ versus $Z^{∕}$ plot, one at a lesser frequency and one at a greater frequency. The semicircle in the lower frequency range denotes the amount of resistance present at the grain boundaries, whereas the second semi-circular arc represents the amount of resistance for each grain [60]. Sometimes there might have some compressed, small and narrow semicircular arc at very lower frequency region as an impact of electrode dominance or interfaces dominance. Fig. 13(a) depicts the Nyquist plot for inspecting compositions from 20 to 100 MHz.

It can be seen that all of the compositions have a clearly distinguishable single semicircular arc with a tendency of second semicircular arc. However, the electrode dominating effect results in a compressed semicircular arc for *x* = 0.0070 and just a little widened one for *x* = 0.0075. Wider semicircular arc at higher frequency might have been caused due to grain resistance and capacitance effect. Thus, the electrical properties of BDTMO can be represented by an equivalent RC circuit as shown in Fig. 13(b). The values of grain resistance (R_g_) and grain boundary resistance (R_gb_) have been calculated from the intercepts on the real axis (Z^/^ axis). The grain capacitance (C_g_) and grain boundary capacitance (C_gb_) have been also estimated. The values of these parameters are listed in Table 4.

**Table 4:** The values of R_g_, R_gb_, C_g_ and C_gb_ of Ba_1-x_Dy_2x/3_Ti_0.98_Mn_0.02_O_3_ ceramics with different *Dy* (x) content.

Sample	*x = 0.0*	*x = 0.007*	*x = 0.0075*	*x = 0.008*	*x = 0.0085*
R_g_ (Ω)	3.01	800	473	1.23	5.51
C_g_ (μF)	0.150	0.143	8.97	0.167	0.610
R_gb_ (MΩ)	56.00	35.20	99.40	60.10	27.60
C_gb_ (μμF)	0.167	0.677	0.549	0.367	0.487

### 3.13 M − H hysteresis loops

**Fig. 14:**
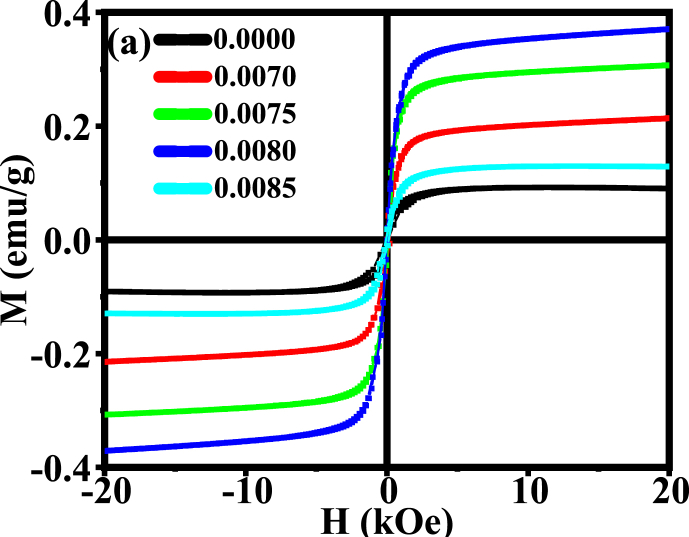
The Magnetic Hysteresis (M − H) loop for various BDTMO ceramics.

The magnetic properties of BDTMO for each sample have been characterized by the physical properties measurement system (PPMS) technique. Fig. 14 shows the magnetization vs magnetic field intensity (M − H) curve, and the associated properties, such as the saturation magnetization (*M*_*s*_), the coercivity (*H*_*c*_) were obtained from the loop which have been given in Table 5. The M − H results indicate that inspecting materials of this series shows weak ferromagnetic nature (nonlinearity of M − H loop) having weak coercive field and small remnant magnetization. The ferromagnetic nature is found to be increased gradually with the substitution of *Dy* till *x* = 0.0080. These findings are consistent with the previous report published elsewhere [37]. The spacing of cations between the A and B positions affects the magnetization. The spins in both positions are antiferromagnetically coupled, resulting in a characteristic magnetic moment that is essentially the numerical difference between the sublattice magnetizations (M_A_-M_B_) [61]. Among all the rare earth elements *Dy* is diffused in both A-site and B-site in barium titanate [62]. The magnetic *Mn* ions are diffused in the B-site [63]. *Dy* has magnetic moment nearly 10*μ*_*B*_, while *Ba* has 0*μ*_*B*_ and *Mn* has 4.5 μ_B_. Hence, increasing the values of *M*_*S*_ causes an increase in *Dy*, which is due to the *Dy* diffusion of A site, resulting an increase of *M*_*A*_*-M*_*B*_. Besides, when *Dy* ion is added in the Ba-site and Mn is added in the Ti-site simultaneously, the overall magnetization increases because of exchange coupling between Mn^2+^ and Ti^4+^ vacancies and Dy^3+^-O^2-^-Dy^3+^ interactions. However, for 0.0085 *Dy* content, falling of *M*_*s*_ is an indication of the coexistence of both ferromagnetism and anti-ferromagnetism nature. The value of *M*_*s*_, however, is greatly affected by the chemical composition, grain size, crystal size, porosity, and density. The increasing Ms with Dy content might also be attributed to the increment of magnetic moment and domain size of the samples [64].

**Table 5:** The variation of M_s_, H_c,_ and *K* with *Dy* of Ba_1-x_Dy_2x/3_Ti_0.98_Mn_0.02_O_3_ ceramics.

Content (x)	(M_s_) emu/g	(H_c_) kOe	K (kOe/emu/g)
0.0000	0.0905	107.3915	10.1238
0.0070	0.2140	20.0711	4.4742
0.0075	0.3060	0.8117	0.2587
0.0080	0.3703	6.0748	2.3432
0.0085	0.1296	60.2864	8.1386

The magnetic properties of the prepared Ba_1-x_Dy_2x/3_Ti_0.98_Mn_0.02_O_3_ composition is compared to earlier several findings [[65], [66], [67], [68], [69]]. It is interesting to point out here that the present solid solution exhibits better results than the other previously reported ceramics. The best values of the reported compositions are presented in Table 6.

**Table 6:** Comparison of the maximum value of *M*_*s*_ with the present investigation.

Ceramics	M_s_ (emu/g)	Refs.
BaTiO_3_	4.2968 × 10^−5^	[67]
BaTi_0.90_Mn_0.10_O_3_	0.013	[37]
Ba_0.99_Sm_0.01_TiO_3_	0.0186	[69]
BaTi_0.25_Mn_0.75_O_3_	1.9866 × 10^−4^	[67]
Ba_0.99_Dy_0.01_Ti_0.95_Fe_0.05_O_3_	0.308	[65]
Ba_0.9920_Dy_0.0053_Ti_0.98_Mn_0.02_O_3_	0.3703	Present work

The anisotropy constant (K) of various Ba_1-x_Dy_2x/3_Ti_0.98_Mn_0.02_O_3_ ceramics was calculated using the relation [68]: $H_{c}=\frac{0.96\times K}{M_{s}}$, where, K is the anisotropy constant, $H_{c}$ is the coercivity, and $M_{s}$ is the saturation magnetization. The variation of K with *Dy* content is listed in Table 5. The anisotropy constant is an important parameter of a magnetic material and the above equation signifies that small anisotropy element has a greater magnetic performance. As shown in Table 5, the undoped sample (*x* = 0.0000) has a higher K value than the doped samples. The highest value of K was obtained for *x* = 0.0085 compound, and this sample comprises the smaller grain size among the studied samples (shown in Fig. 5(b)). The doped compositions have lower K values, which contribute to the increase of their magnetic characteristics.

### 3.14 Initial permeability measurement

**Fig. 15:**
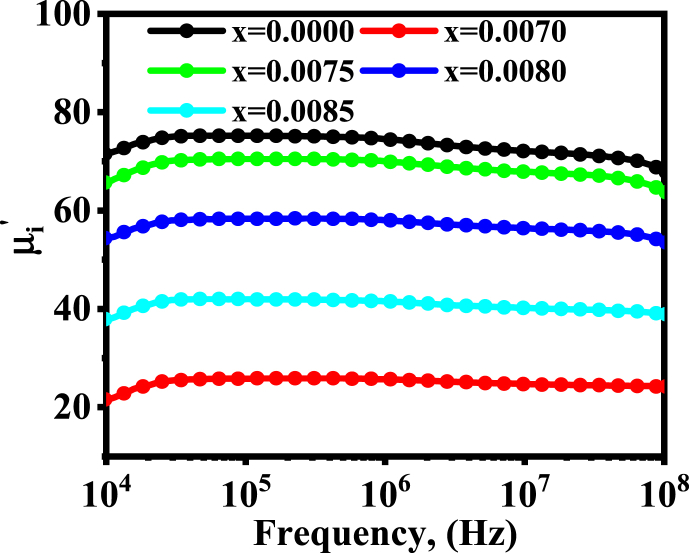
The variations in $\mu_{i}^{/}$ with frequency for different BDTMO ceramics.

The complex initial permeability is given by ${\;\mu }_{i}^{*}=\mu_{i}^{/}-{i\mu }_{i}^{∕∕}$, where $\mu_{i}^{/\;}$ is the real part of the complex permeability and $\mu_{i}^{∕∕}$ is the imaginary part of the complex permeability. The field-dependent $\mu_{i}^{*}$ offers information about the domain walls' inertial state, changing behavior, and mutual coupling. Fig. 15 displays the frequency dependency of the real part of initial permeability. Samples with *x* = 0.0000 have a maximum value of $\mu_{i}^{/}$ within the studied frequency range and, then, there is a decrease in $\mu_{i}^{/}$ with the substitution of *Dy*^*3+*^ content. However, the decreasing pattern is not gradual, the minimum value of $\mu_{i}^{/}$ is for *x* = 0.0070, and the highest value is obtained for *x* = 0.0075. The parameters, which have an effect on the increment and decrement of $\mu_{i}^{/}$, are dependent on the average size of the particle, their distribution in the material, and also on the magnetic and electrical parameters. The most influential parameters on $\mu_{i}^{/}$ are the degree of spontaneous magnetization and the coefficient of magnetic crystallographic anisotropy [70]. Beside these, the permeability also depends on many parameters, such as composition, stoichiometry, impurity content, average crystallite size, grain size distribution, porosity, crystalline anisotropy, so on. The increment of permeability is a consequence of increasing crystallite size and cationic stoichiometry, decreasing porosity, and also on small crystallographic anisotropy constant [71].

In the present study, the grain size has been found to be increased and *P (%)* has been decreased, *M*_*s*_ has been increased, yet, there is a decreasing pattern of permeability out of increment after substituting of *Dy*^*3+*^ ion. The cause might go to the increase of monocrystalline anisotropy. Since the permeability, resulting from the domain wall motion, is given by equation (13) [72],

(13) $$\mu ‐1=3\pi \;M_{S}^{2}D/4\gamma$$

where, *M*_*S*_ represents the saturation magnetization, *D* represents the grain size, and *γ* is the domain wall energy, which is proportional to the crystallographic anisotropy shown in equation (14). Therefore,

(14) μ ≈ M_S_^2^D/K

Which indicates that if the magneto crystalline anisotropy increases, the permeability will decrease. Another cause of decreasing the permeability might be due to the shift of *Ba*^*2+*^*-O- Ba*^*2+*^exchange interaction to *Ba*^*2+*^*-O-Dy*^*3+*^ resulting from the crystallographic anisotropy. The main contributor to decrease the permeability may be due to the barrier against the spinning rotation and the domain wall motion caused by the suppression of some contaminants or oxygen vacancies inside the sample [73]. Finally, it can be mentioned that the permeability may have a tendency to decrease, but the stability of the sample is much more obvious. This is because the permeability remains constant throughout a broad range of frequencies, which is actually expected for the mechanical and microwave applications.

### 3.15 Magnetic loss factor tan*δ*_*M*_ and relative quality factor RQF

**Fig. 16:**
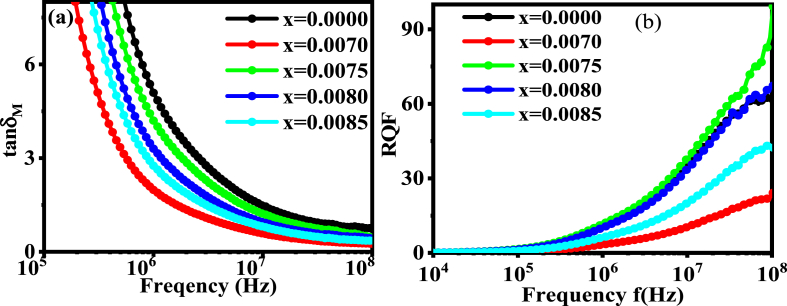
The variation of (a) tan*δ*_*M*_ and (b) RQF for *Dy* into BDTMO ceramics.

Fig. 16(a) shows the magnetic loss tangent (tan*δ*_*M*_*)* as the dependency of frequency for all samples. The figure shows that the loss factor decreases exponentially with increasing frequency. Such characteristic is attributed due to creation of barrier at high frequency against the spontaneous domain wall motion [74]. It is also obvious by inspecting Fig. 16(a), the compositions dopped with *Dy* have a lower tan*δ*_*M*_ value. For most magnetic applications, like microwave devices, smaller values of loss factor at higher frequencies is required.

The variation of relative quality factor (RQF) with frequency of the prepared compound is shown in Fig. 16(b). The RQF goes up as the frequency increases. Earlier reports mentioned that the monotonously increasing RQF reaches a maximum at a given frequency, and the deterioration occurs as a result of an abrupt drop in the domain wall motion with respect to the applied magnetic field [75]. As shown in Fig. 16(b), the values of Q-factor of *Dy* replaced by perovskite are found slightly lower than that of parent sample *x* = 0.0000, except the one observed for *x* = 0.0075 *Dy* doping. This could be owing to the sample's increased hysteresis loss, which enhance with porosity [76].

## 4 Conclusions

In the present investigation, the polycrystalline Ba_1-x_Dy_2x/3_Ti_0.98_Mn_0.02_O_3_ (BDTMO) ceramics were fabricated using the traditional solid phase reaction method. The influences of co-doping of *Dy* (low amount) and Mn on the crystal symmetry, microstructure, dielectric, electric, and magnetic properties of the compositions were investigated. The *Dy* dopped BDTMO ceramics' XRD patterns displayed a nicely ordered crystalline tetragonal structure. There was no evidence of secondary phase or impurities in the samples’ XRD patterns. The value of *ρ*_*B*_ was found to be raised up to *x* = 0.0080 and, then, experienced a decrease, whereas the porosity exhibited the opposite trend. A significant increase in the average size of grains ($\bar{D}$) was observed when *Dy* was dopped, but the $\bar{D}$ value decreased as a result of the subsequent rise in *Dy* concentration. The highest magnitude of $\bar{D}$ (0.5713 μm) was obtained for the compound *x* = 0.0070. All of the compositions showed the typical low-frequency dielectric dispersal behavior due to the Maxwell-Wagner interfacial polarization. The maximum magnitude of $\varepsilon^{/}$ was found more than 6000 at 10^3^ Hz for the *x* = 0.0070 sample. The tanδ_E_, $Z^{/}$, and $Z^{∕∕}$ were also demonstrated similar trends, a decreasing propensity with increasing the frequency and also remained almost independent in the higher-frequency region. A very small value of tanδ_E_ (<5 %) is noticed for *x* = 0.0070 sample. The values of R_g_ and C_g_ were determined from the Nyquist plot and it suggested that the grain boundary played a significant role in the conductivity mechanism of the material being studied. The M − H hysteresis loops confirmed a rather weak ferromagnetic nature of the compound, where the saturation magnetization (*M*_*S*_) has increased consistently up to *x* = 0.0080 level of *Dy*; however, it was then showed a sudden fall. The optimal value of *M*_*S*_ 0.3703 emu/g was estimated for the *x* = 0.0080 solid solution. For the sample with *x* = 0.0075, the best value of $\mu_{i}^{/}$ was found to be 69. It is obvious to note that the compound with *x* = 0.0070 possessed relatively bigger size grains, the maximum dielectric constant, and the minimum dielectric loss. The composition with *x* = 0.0080 has the highest value of *ρ*_*B*_ (5.4618 g/cm^3^) and has a better magnetic coupling capability. The synthesized compound is a lead-free BaTiO_3_-based ceramics, which is superior to the other perovskite elements, available in the local market. It is environmentally friendly, and offers the best value of saturation magnetization and an excellent stability of frequency*.* Because of improved quality and superior property, the fabricated material is a great choice for the use in both magnetic and electric devices, such as energy storage devices, multifunctional sensors, magnetoelectric memory cells, and multilayer capacitors. The finding is particularly important due to the fact that very small amounts of *Dy* doping could bring a significant modification in the electrical and magnetic properties of BaTiO_3_. Moreover, the manufactured compound is economically feasible, offers a low production cost.

## Data availability statement

No. The data that has been used is confidential.

## Ethics declarations

Review and/or approval by an ethics committee was not needed for this study because it is an experimental work in the field of material science.

## CRediT authorship contribution statement

**S.A. Mamun:** Writing – original draft, Methodology, Investigation, Conceptualization. **Mithun Kumar Das:** Writing – review & editing. **K.S. Uddin:** Methodology, Investigation, Data curation. **T. Ahamed:** Writing – review & editing. **Mohammad J. Miah:** Writing – review & editing, Supervision, Project administration.

## Declaration of competing interest

The authors declare that they have no known competing financial interests or personal relationships that could have appeared to influence the work reported in this paper.
